# Supplementary material for: The multinomial index: a robust measure of reproductive skew
Source: Proc Biol Sci. 2020 Oct 7;287(1936):20202025. doi: 10.1098/rspb.2020.2025 (PMC7657858; doi:10.1098/rspb.2020.2025)
Supplement: Appendix [file rspb20202025supp1.pdf]

Supplementary Information to:
The Multinomial Index: A Robust Measure of Reproductive Skew
In: *Proceedings of the Royal Society B*

DOI: 10.1098/rspb.2020.2025

Cody T. Ross[a], Adrian V. Jaeggi[b], Monique Borgerhoff Mulder[c], Jennifer E. Smith[d], Eric Alden Smith[e], Sergey Gavrilets[f], Paul L. Hooper[g]

[a] *Department of Human Behavior, Ecology and Culture. Max Planck Institute for Evolutionary Anthropology. Leipzig, Germany.*
[b] *Institute of Evolutionary Medicine. University of Zurich. Zurich, Switzerland.*
[c] *Department of Anthropology. University of California, Davis. Davis, CA. USA.*
[d] *Department of Biology. Mills College. Oakland, CA. USA.*
[e] *Department of Anthropology. University of Washington. Seattle, WA. USA.*
[f] *Departments of Ecology & Evolutionary Biology and Mathematics. University of Tennessee, Knoxville. Knoxville, Tennessee. USA.*
[g] *Santa Fe Institute. Santa Fe, NM. USA.*

Contents

1 Derivation 1

2 Estimation of age-specific RS 2
- 2.1 Elasticity model 2
- 2.2 Gaussian Process model 2
  - 2.2.1 The model 3
  - 2.2.2 Testing with simulated data 3

3 Bayesian estimation of M 4

4 SkewCalc details 4

5 Model fit diagnostics, empirical models 4

6 Model fit diagnostics, simulation models 4

7 Bayesian phylogenetic mixed-effects models 5
- 7.1 Model specification 5
- 7.2 Phylogenetic tree 5
- 7.3 Model fit diagnostics 5

8 Full K&N reanalysis results 5
- 8.1 Univariate associations 5
- 8.2 Multivariate associations 6
- 8.3 Intra-specific analysis 6

1. Derivation

Again, we note that N is the number of individuals in the sample, R is the total offspring produced by all in-sample individuals, T is the total exposure time contributed by all in-sample individuals, r_i is the number of offspring produced by individual i, t_i is the exposure time of individual i, $\bar{r}_i = \frac{R}{T} t_i$ is the expected number of offspring that individual i would have produced if RS rates were equal across all individuals in the population, $\hat{r}_i = \frac{r_i}{R}$ is the share of reproduction held by individual i, and finally $\hat{t}_i = \frac{t_i}{T}$ is the share of exposure time contributed by individual i. We begin with the definition of $\check{M}(r,t)$ introduced in the main text:

$$\check{M}(r,t) = \frac{N^2}{R^2} \frac{1}{N} \sum_{i=1}^{N} (r_i - \bar{r}_i)^2 \tag{1a}$$

$$= \frac{N^2}{R^2} \frac{1}{N} \sum_{i=1}^{N} (r_i - \tfrac{R}{T} t_i)^2 \tag{1b}$$

$$= \frac{N^2}{R^2} \frac{1}{N} \sum_{i=1}^{N} (r_i - R\hat{t}_i)^2 \tag{1c}$$

Eq. 1(c) can then be expanded as follows (by linearity of finite sums):

$$\check{M}(r,t) = \frac{N^2}{R^2} \Big[\frac{1}{N} \sum_{i=1}^{N} (r_i)^2 - 2\frac{1}{N} \sum_{i=1}^{N} (r_i \hat{t}_i R) + \frac{1}{N} \sum_{i=1}^{N} (R\hat{t}_i)^2 \Big] \tag{2}$$

Next, we note that the definition of a variance (McElreath and Boyd, 2008, pp. 335) is: $\text{var}(Y) = \mathbb{E}[Y^2] - \mathbb{E}[Y]^2$, and the definition of a covariance (McElreath and Boyd, 2008, pp. 336) is: $\text{cov}(Y,X) = \mathbb{E}[YX] - \mathbb{E}[Y]\mathbb{E}[X]$. So, we can write Eq. 2 in a more compressed notation as:

$$\check{M}(r,t) = \frac{N^2}{R^2} \Big[(\text{var}(r) + \tfrac{R^2}{N^2}) - 2(\text{cov}(r, R\hat{t}) + \tfrac{R^2}{N^2}) + (\text{var}(R\hat{t}) + \tfrac{R^2}{N^2}) \Big] \tag{3a}$$

$$= \frac{N^2}{R^2} \Big[\text{var}(r) + \text{var}(R\hat{t}) - 2\text{cov}(r, R\hat{t}) \Big] \tag{3b}$$

Now, we note from McElreath and Boyd (2008, pp. 336) that the slope of the regression line of Y on X is:

$\beta(Y,X) = \frac{\mathrm{cov}(Y,X)}{\mathrm{var}(X)}$. So we can write Eq. 3(b) as:

$$\check{M}(r,t) = \frac{N^2}{R^2}\left[\mathrm{var}(r) + \mathrm{var}(R\hat{t}) - 2\beta(r, R\hat{t})\mathrm{var}(R\hat{t})\right] \quad (4)$$

Here, we note the assumption that the conditional expected value of r given normalized exposure time $\hat{t}$ is: $\mathbb{E}[r_i|\hat{t}_i = x] = Rx$. In other words, we assume that reproductive success, r, is on average proportional to exposure time t with a proportionality constant equal to the mean RS rate, $\frac{R}{T}$: $r \sim \frac{R}{T}t$, or equivalently: $r \sim R\hat{t}$. This means that we can evaluate Eq. 4 when: $\beta(r, R\hat{t}) = 1$. This yields:

$$\check{M}(r,t) = \frac{N^2}{R^2}\left[\mathrm{var}(r) + \mathrm{var}(R\hat{t}) - 2\mathrm{var}(R\hat{t})\right] \quad (5a)$$

$$= \frac{N^2}{R^2}\left[\mathrm{var}(r) - \mathrm{var}(R\hat{t})\right] \quad (5b)$$

Eq. 5(b) indicates that $\check{M}(r,t)$ can be understood as a normalized variance in RS after subtracting out the variance in RS owed to variance in exposure time to risk of RS.

In fact, factoring out $\mathrm{var}(r)$ from both terms in brackets in Eq. 5(b), yields Eq. 6(a). Then, noting that $\frac{\mathrm{var}(\mathbb{E}[Y|X])}{\mathrm{var}(Y)} = \mathrm{corr}(Y,X)^2$ (see Bowsher and Swain, 2012, SI pp. 3-4), and that $\mathrm{var}(\mathbb{E}[r|\hat{t}]) = \mathrm{var}(R\hat{t})$, we see that:

$$\check{M}(r,t) = \frac{N^2}{R^2}\left[\mathrm{var}(r)\left(1 - \frac{\mathrm{var}(R\hat{t})}{\mathrm{var}(r)}\right)\right] \quad (6a)$$

$$\check{M}(r,t) = \frac{N^2}{R^2}\left[\mathrm{var}(r)\left(1 - \mathrm{corr}(r, R\hat{t})^2\right)\right] \quad (6b)$$

Here, $\mathrm{corr}(Y,X)^2$ is the coefficient of determination, which measures the fraction of variance in Y explained by the regression of Y on X. Eq. 6(b) therefore shows that $\check{M}(r,t)$ can be understood as the standardized residual fraction of variance in r that is not explained by the (slope=1) regression of observed RS on the RS expected given the observed exposure time data and an assumption of equal RS rates.

Finally, the "law of total variance" (Blitzstein and Hwang, 2019, pp. 434) states that: $\mathrm{var}(Y) = \mathbb{E}[\mathrm{var}(Y|X)] + \mathrm{var}(\mathbb{E}[Y|X])$. Translating this into our variables, we have: $\mathrm{var}(r) = \mathbb{E}[\mathrm{var}(r|\hat{t})] + \mathrm{var}(\mathbb{E}[r|\hat{t}])$. Slight rearranging, yields: $\mathbb{E}[\mathrm{var}(r|\hat{t})] = \mathrm{var}(r) - \mathrm{var}(R\hat{t})$. So $\check{M}$ can be understood as a standardized conditional variance:

$$\check{M}(r,t) = \frac{N^2}{R^2}\left[\mathrm{var}(r) - \mathrm{var}(R\hat{t})\right] \quad (7a)$$

$$= \frac{N^2}{R^2}\left[\mathbb{E}[\mathrm{var}(r|\hat{t})]\right] \quad (7b)$$

2. Estimation of age-specific RS

2.1. Elasticity model

The basic derivation of M assumes that the risk of reproduction is independent of age—or equivalently, that all years in the life course contribute equally to risk of reproductive success. In some populations, especially human populations, however, this assumption is likely to be violated. If we assume that the relationship between age and expected reproductive success over a time interval can be measured using an elasticity parameter, $\beta \in (0,1)$, where:

$$\mathbb{E}[r_i|a_i, b_i] = \alpha(b_i^\beta - a_i^\beta) \quad (8)$$

then we can write a more general definition for $\check{M}(r,t)$ as $\check{M}(r,a,b,\beta)$, where a_i is the age of individual i at first observation and b_i the age at death or censor. This is:

$$\check{M}(r,a,b,\beta) = N\sum_{i=1}^{N}\left(\hat{r}_i - \frac{b_i^\beta - a_i^\beta}{\sum_{j=1}^{N}(b_j^\beta - a_j^\beta)}\right)^2 \quad (9)$$

Note that Eq. 9 reduces to the original expression for $\check{M}$ given in the main text as $\beta \to 1$, since $b_i^1 - a_i^1$ is by definition the same quantity as t_i. In other words, when there are no diminishing returns to age, then $\beta = 1$ and $\check{M}(r,t) = \check{M}(r,a,b,\beta)$. In the basic formulation of $\check{M}$, if reproductive rates were equal, the expected number of offspring produced by individual i would be equal to $\bar{r}_i = R\hat{t}_i = \frac{R}{T}t_i$. In the generalized version of $\check{M}$, if reproductive rates were equal across all individuals within an age-class, but the value of each year of life to the production of offspring declines with age, then we instead have $\bar{r}_i = R\frac{b_i^\beta - a_i^\beta}{\sum_{j=1}^{N}(b_j^\beta - a_j^\beta)}$. Each individual's share of exposure time is adjusted to account for diminishing returns to age, and then these age-adjusted exposure times are normalized to yield a share parameter. Empirically, the parameters of the model—α and β—can be estimated on a population-by-population basis using the same data needed to estimate M itself.

2.2. Gaussian Process model

In many cases, the assumption that the conditional expected value of RS is proportional to exposure time is problematic. In the main paper, we provide an initial relaxation of this restrictive assumption by assuming that there may be decreasing marginal returns to age. There, we derive a more general version of $\check{M}$ that uses an elasticity parameter to estimate and account for variation in RS introduced due to diminishing marginal returns to age. However, for some wildly non-linear age-specific RS curves, even this approach is insufficient to provide a good measure of the conditional expected value of RS given a set of exposure time data.

Here, we derive a substantially more general—but also more computationally expensive—approach that uses a Gaussian Process (GP) model (Stan Development Team, 2019, pp. 136–146) to estimate the actual functional form of the expected age-specific RS curve. We define such a model below, provide a function to fit such a model in our `SkewCalc` package, and provide some comparisons of this metric's performance versus the simpler models using simulated data. We show that the GP model is usually more widely applicable, but we note that this comes with the trade-offs of being computationally slower to implement and requiring a larger sample size to effectively estimate.

2.2.1. The model

Let there be K total age classes, and then let logisitic($\phi_{[k]}$)—which has a lower limit at 0, and an upper limit at 1—be the effective exposure-time contribution of age class k, then:

$$\mathbb{E}[r_{[i]}|a_{[i]}, b_{[i]}] = \omega \sum_{k=(a_{[i]}+1)}^{b_{[i]}} \text{logisitic}(\phi_{[k]}) \tag{10}$$

where ω is an RS rate per unit effective exposure time, and where $a_{[i]} \in \mathbb{N}$ is the age of individual i at time of first observation ($a_{[i]} = 0$ if an individual is observed from birth) and $b_{[i]} \in [\mathbb{N} > a_{[i]}]$ is the age of individual i at death or censor.

We then estimate ϕ using a Gaussian process:

$$\phi = \mu + \sigma \text{L}(\Omega)\hat{\phi} \tag{11}$$

where μ is an intercept parameter, σ is a standard deviation parameter, $\hat{\phi}$ is a vector of unit-normal random effects, and L(Ω) gives a factor from the Cholesky decomposition of the correlation matrix Ω.

The parameters μ and σ have weak priors:

$$\mu \sim \text{Normal}(0, 1) \tag{12}$$

$$\sigma \sim \text{Exponential}(1) \tag{13}$$

and the correlation matrix, Ω, is defined by:

$$\Omega_{[i,j]} = \rho \exp\Big(- \xi \frac{(i-j)^2}{K^2} \Big) \tag{14}$$

where the priors are generally weak, but assume that adjacent age categories have correlated effective exposure time contributions:

$$\rho \sim \text{Beta}(12, 2) \tag{15}$$

$$\xi \sim \text{Exponential}(1) \tag{16}$$

2.2.2. Testing with simulated data

Fig. 1 plots our tests with simulated data. In frame (a) we plot simulated RS by age data. We generate simulated RS data as a function of age using three models (proportional returns, diminishing returns, and highly non-linear returns) for each of two skew values (no skew—based on Poisson residuals, and high skew—based on over-dispersed, Negative Binomial residuals). Orange points are unweighted years lived. Black points are effective years lived after value-weighting age/exposure time using the age-specific RS functions in frames (b) and (c). When RS values are generated such that they are proportional to age (left column), then value weighting has no effect on the structuring of variation in exposure time (but note that points are jittered for clarity). However, when there are diminishing returns to age (middle column) or radically non-linear returns to age (right column), then value-weighting years-lived can change how variation in exposure time and thus RS is structured.

Frames (b) and (c) show three age-specific RS functions—either cumulative RS (left column) or instantaneous RS per year (right column). The first row shows RS being proportional to age. The second row shows diminishing RS returns to age. The final row shows a highly non-linear function linking RS and age; in this case, there are two periods of fertility separated by a period of infertility. The black curves in frames (b) and (c) are the curves used to generate the simulated data; these curves were identical in the generative model of RS without skew (frame b) and with skew (frame c). Posterior estimates of these same curves are plotted using elasticity control (in purple) and GP control (in orange) for each data set. Because exposure time data is only modeled up to a proportion, only the shape, not the y-axis location of the function should be evaluated here.

If RS is proportional to age, then the basic M metric, the M metric with elasticity control, and the M metric with GP control, all recover the correct form of the value function, and generate equivalent estimates in frame (d). If marginal RS is diminishing with age, then the basic M metric is upwardly biased, but the M metric with either elasticity control or GP control recovers the correct form of the value function and produces correct estimates in frame (d). If RS is a highly non-linear function of age (as it is in the two-phase function plotted in Fig. 1), then both the standard M metric and the M metric with elasticity control fail to recover the correct form of the value function; the M metric with GP control, however, still recovers the correct form of the value function and produces correct estimates in frame (d).

Frame (d) shows that estimates of M with GP control behave correctly under each simulation model, as is indicated by the locations of the density distributions being invariant to the age-specific RS function used to generate the data; the "no skew" density distributions include 0 (the value that M should take when residual RS variance conditional on effective exposure time is Poisson distributed), and the "skew" density distributions are > 0 (the value that M should take when residual RS variance conditional on effective exposure time is over-dispersed relative to a Poisson distribution). Estimates of M based on elasticity control work well if the age-specific RS function is either proportional to age or exhibits diminishing marginal returns to age. Estimates of M based on the simple proportionality assumption (i.e., the most basic formulation of M) only work well if age-specific RS is actually proportional to age.

[Figure 1 about here.]

3. Bayesian estimation of M

Both $\check{M}$ and M as expressed in the main text are point estimates that do not reflect the uncertainty inherent in their calculations. When N is small, for example, estimates are less credible; downstream analysis of skew indices should account for this uncertainty.

To estimate the posterior probability distributions of $\check{M}$ and M given a specific data set, we first define the posterior densities corresponding to $\hat{t}$, which we will denote here as $\tilde{t}$. The posterior of $\tilde{t}$ can be defined a few different ways: not controlling for variable returns to age (Eq. 17), controlling for variable returns to age using an elasticity parameter (Eq. 18), or controlling for variable returns to age using the GP method just described (Eq. 19):

$$\tilde{t}_{[i]} = \frac{t_{[i]}}{\sum_{j=1}^{N} t_{[j]}} = \frac{b_{[i]} - a_{[i]}}{\sum_{j=1}^{N} \left(b_{[j]} - a_{[j]}\right)} \tag{17}$$

$$\tilde{t}_{[i]} = \frac{b_{[i]}^{\beta} - a_{[i]}^{\beta}}{\sum_{j=1}^{N} \left(b_{[j]}^{\beta} - a_{[j]}^{\beta}\right)} \tag{18}$$

$$\tilde{t}_{[i]} = \frac{\sum_{k=(a_{[i]}+1)}^{b_{[i]}} \text{logisitic}(\phi_{[k]})}{\sum_{j=1}^{N} \sum_{k=(a_{[j]}+1)}^{b_{[j]}} \text{logisitic}(\phi_{[k]})} \tag{19}$$

While $a_{[i]}$ and $b_{[i]}$ (or, alternatively, $t_{[i]}$) will often be data, they can just as easily be parameters reflecting missing data or partially known data (i.e., data measured with error). The parameter β, if used, should have a weak, positive-constrained prior, like: $\beta \sim \text{Folded-Normal}(0, 1)$.

Next, we define the posterior density corresponding to $\hat{r}$, which we will denote here as $\tilde{r}$. We give $\tilde{r}$ a Dirichlet prior: $\tilde{r} \sim \text{Dirichlet}(\tilde{t}\kappa)$, where $\tilde{t}\kappa$ gives weak prior support in proportion to exposure time, and κ itself has a weak prior: $\kappa \sim \text{Log-Normal}(0, 2.5)$.

Then, we model the generative process of reproductive outcomes:

$$r \sim \text{Multinomial}(R, \tilde{r}) \tag{20}$$

These models are fit using a variant of Hamiltonian Monte Carlo via `Stan` (Stan Development Team, 2017), which samples fast and efficiently even for large outcome vectors.

The posterior distributions of the skew indices are then calculated by applying the relevant function to the relevant parameter vectors at each Monte Carlo sample.

4. SkewCalc details

We provide an `R` package (R Core Team, 2015)—`SkewCalc`—that can calculate point estimates for the full set of functions described in this paper: $\check{M}(r, t)$ as `Mraw_index(r,t)`, $\check{M}(r, a, b)$ as `Mraw_index_age(r,t,t0)`, $M(r, t)$ as `M_index(r,t)`, and $M(r, a, b)$ as `M_index_age(r,t,t0)`. Additionally, we provide a function for Nonacs' B as `B_index(r,t)`, and functions that can convert Nonacs' B values into estimated M values, `Mraw_index_from_B_index(B,R,N)` for the unadjusted variant and `M_index_from_B_index(B,R,N)` for the adjusted variant. Additionally, the `R` package includes a Bayesian model, fit using Stan (Stan Development Team, 2017), that can estimate the posterior densities of the above-listed measures from individual-level RS and exposure time data: `M_index_stan(r,t,t0)`. Each function is documented with example tests, and further details can be acquired by calling the function name with a `?` in `R`: e.g., `?M_index_stan`.

The `R` package is installed with two lines of code:

```
library(devtools)
install_github("Ctross/SkewCalc")
```

Further information about the `SkewCalc` package, including a set of example workflows, is available at https://github.com/ctross/SkewCalc. Bug-reports, feature requests, and other relevant comments should be made through GitHub, where the package will be maintained.

5. Model fit diagnostics, empirical models

To check the fit of the Bayesian model on the empirical data used to make Figure 2 of the main text, we check traceplots, effective sample size, and r-hat. Traceplots (Figures 2, 3, and 4) generally indicate good MCMC behavior—thorough mixing and apparent convergence of multiple chains to the same posterior region. Estimated effective sample size is greater than 300 across all models and parameters, and r-hat values are less than 1.02 across all models and parameters.

[Figure 2 about here.]

[Figure 3 about here.]

[Figure 4 about here.]

6. Model fit diagnostics, simulation models

To check the fit of the Bayesian model on the simulated data used to make Figure 3 of the main text, we check traceplots, effective sample size, and r-hat. Traceplots (Figure 5) indicate good MCMC behavior for a sample of the models fit in Figure 3 of the main text. We observe thorough mixing and apparent convergence of multiple chains to the same posterior region.

[Figure 5 about here.]

7. Bayesian phylogenetic mixed-effects models

7.1. Model specification

Since phylogenetic methods and Markov Chain Monte Carlo software have significantly improved in recent years, we use newly developed phylogenetic mixed-effect models (de Villemereuil and Nakagawa, 2014) implemented in Stan (Stan Development Team, 2017) to conduct a secondary analysis of the K&N data. We use Stan templates produced via `brms` (Bürkner, 2018) as a starting point, and then extend the basic `Stan` models generated with `brms` to deal with missing data.

Let some skew metric Z in population j have the distribution:

$$Z_{[j]} \sim \text{Normal}(\mu_{[j]}, \sigma) \tag{21}$$

we then model:

$$\mu_{[j]} = \alpha + \rho_{[S(j)]} + \gamma_{[S(j)]} + \dot{\beta}_{[1]}\bar{X}_{[j]} + \ldots + \ddot{\beta}_{[1]}(X_{[j]} - \bar{X}_{[j]}) + \ldots \tag{22}$$

where α is an intercept, $S(j)$ is a function giving the species index of population j, ρ is a vector of species-specific random effects incorporating phylogenetic control, γ is a vector of species-specific random effects not incorporating phylogenetic control, $\dot{\beta}$ are the set of regression coefficients for the between-species effects, and $\ddot{\beta}$ are the set of regression coefficients for the within-species effects. We denote the mean value of some covariate X in the species of population j as $\bar{X}_{[j]}$, and the population-specific offset from this species mean as $(X_{[j]} - \bar{X}_{[j]})$.

The parameters α, $\dot{\beta}$, and $\ddot{\beta}$ have simple priors:

$$\alpha \sim \text{Normal}(0, 5); \tag{23}$$

$$\dot{\beta} \sim \text{Normal}(0, 5); \tag{24}$$

$$\ddot{\beta} \sim \text{Normal}(0, 5); \tag{25}$$

The priors on the parameter vector γ have a slightly more complex, multi-level structure:

$$\gamma \sim \text{Normal}(0, \sigma_\gamma); \tag{26}$$

The priors on the parameter vector ρ have a still more complex, multi-level structure:

$$\rho \sim \text{Multivariate Normal}((0, \ldots, 0)', \Omega); \tag{27}$$

where:

$$\Omega = \text{Diag}(\sigma_\rho)\Psi\text{Diag}(\sigma_\rho) \tag{28}$$

and where Ψ, a correlation matrix, is defined using the normalized phlyogenetic distance $D_{[i,j]}$ between groups i and j as:

$$\Psi_{[i,j]} = \exp(-\zeta D_{[i,j]}) \tag{29}$$

A positive-constrained prior is given to ζ:

$$\zeta \sim \text{Folded Normal}(0, 1) \tag{30}$$

Likewise, positive-constrained priors are given to σ, σ_γ, and σ_ρ:

$$\sigma \sim \text{Folded Cauchy}(0, 1) \tag{31}$$

$$\sigma_\gamma \sim \text{Folded Cauchy}(0, 1) \tag{32}$$

$$\sigma_\rho \sim \text{Folded Cauchy}(0, 1) \tag{33}$$

Missing data are treated as parameters (McElreath, 2016). Non-missing, continuous covariate data are first standardized. Then, any missing continuous data points are replaced in the model by parameters with Normal(0,1) prior distributions. Any missing binary data points are replaced in the model by parameters with Beta(1,1) prior distributions.

7.2. Phylogenetic tree

A consensus phylogeny for all species in the sample was downloaded from the 10ktrees website Version 3 (Arnold et al., 2010). For steps 1 and 2, we followed K&N in setting all branch lengths equal (to 1) (Figure 6a) and for our phylogenetic mixed-effects models we used the full tree with numerical branch length (Figure 6b.)

[Figure 6 about here.]

7.3. Model fit diagnostics

To check the fit of the Bayesian model on the empirical data used to make Figure 4 of the main text, we check traceplots, effective sample size, and r-hat. Traceplots of the multivariate models (Figures 7a, 7b, 7c, and 7d) generally indicate good MCMC behavior—thorough mixing and apparent convergence of multiple chains to the same posterior region. Estimated effective sample size in the multivariate models is greater than 900 across all slope parameters, and r-hat values are less than 1.02 across all slope parameters in the multivariate models.

[Figure 7 about here.]

8. Full K&N reanalysis results

Here, we present the full description of the results, including the robustness checks described in the main text.

8.1. Univariate associations

Table 1 presents the univariate associations between different measures of skew and demographic and reproductive variables. The results for λ and MMP are not qualitatively different from Table 1 in K&N, excepting some deviation explained by the use of a slightly different phylogeny and different treatment of outliers. Results for B were not presented by K&N, but are presented here. Using species means, M was moderately correlated with λ (ρ=0.32), and MMP (ρ=0.30), and strongly correlated with B (ρ=0.91), due to the consistently small sample sizes of males.

[Table 1 about here.]

In our analysis, there was no significant association between B or M and male group size. There was, however, a significant association between female group size and B. Since B and M were not available for as many species as λ or MMP, we repeated the analyses after restricting the dataset to species where all measures were available (Table 2). The results from these analyses are not qualitatively different from the main analysis.

[Table 2 about here.]

8.2. Multivariate associations

Table 3 shows the results of multiple regression models testing the associations between different skew indices and other variables (Table 2 in K&N). Here, MMP and B show significant negative relationships with male number. When restricting the sample to species for which all skew indices were available (Table 4), all skew measures except M show a significantly negative effect of male group size on skew. Female group size shows a significant negative relationship with maximum mating proportion and B.

[Table 3 about here.]

[Table 4 about here.]

8.3. Intra-specific analysis

K&N reported step-wise multiple regression models showing significant relationships between male group size and both λ and MMP, and no relationships between the suite of considered skew measures and female group size or expected estrous overlap. We were able to reproduce these results; moreover, our analysis finds no significant relationship between M and male group size, female group size, or expected estrous overlap (Tables 5 and 6), in comparable models.

[Table 5 about here.]

[Table 6 about here.]

Restricting the dataset to cases where all skew indices were available (Tables 7 and 8) did not qualitatively change these results; male number continues to have a statistically significant relationship with λ and MMP. There is a marginally significant relationship with B, but no significant relationship with M.

[Table 7 about here.]

[Table 8 about here.]

Approvals

Regarding the Colombian data, informed consent was obtained from each respondent and the community leader (when appropriate) prior to data collection. Because of limited literacy rates at the study sites, informed consent was obtained verbally. All field protocols were approved by the Max Planck Institute for Evolutionary Anthropology, Department of Human Behavior, Ecology and Culture, and declared exempt from additional IRB oversight.

Regarding the Kipsigis data, permission to conduct research in Kenya was granted by the Office of the President, Nairobi. Informed consent was obtained from each respondent. All field protocols were approved at Northwestern University. Special thanks to the Kipsigis families for their friendship and cheerful cooperation throughout the study.

Acknowledgments

This project was sponsored by the National Institute for Mathematical and Biological Synthesis, supported through National Science Foundation awards EF-0832858 and DBI-1300426, with additional support from The University of Tennessee, Knoxville. CR was supported by the Dynamics of Wealth Inequality project of the Behavioral Sciences Program at the Santa Fe Institute, the United States National Science Foundation (NSF-IBSS grant no. 1329089), and the Max Planck Institute for Evolutionary Anthropology. SG was supported by the U.S. Army Research Office grants W911NF-14-1-0637 and W911NF-17-1-0150, the Office of Naval Research grant W911NF-18-1-0138. We thank Richard McElreath, Dieter Lukas, and the MPI-EVA lab group for detailed feedback on earlier versions of this paper, along with our anonymous reviewers.

References

Arnold C, Matthews LJ, Nunn CL. The 10ktrees website: A new online resource for primate phylogeny. Evolutionary Anthropology 2010;19(3):114–8.

Blitzstein JK, Hwang J. Introduction to Probability. CRC Press, 2019.

Bowsher CG, Swain PS. Identifying sources of variation and the flow of information in biochemical networks. Proceedings of the National Academy of Sciences 2012;109(20):E1320–8.

Bürkner PC. Advanced Bayesian Multilevel Modeling with the R Package brms. The R Journal 2018;10(1):395–411. URL: https://doi.org/10.32614/RJ-2018-017. doi:10.32614/RJ-2018-017.

McElreath R. Statistical Rethinking: A Bayesian Course with Examples in R and Stan. CRC Press, 2016.

McElreath R, Boyd R. Mathematical models of social evolution: A guide for the perplexed. University of Chicago Press, 2008.

R Core Team . R: A Language and Environment for Statistical Computing. R Foundation for Statistical Computing; Vienna, Austria; 2015. URL: https://www.R-project.org/.

Stan Development Team . Stan: A C++ library for probability and sampling, version 2.16.0. 2017. URL: http://mc-stan.org/.

Stan Development Team . Stan Users Guide, Version 2.24; 2019. URL: http://mc-stan.org/.

de Villemereuil P, Nakagawa S. General Quantitative Genetic Methods for Comparative Biology; Berlin, Heidelberg: Springer Berlin Heidelberg. p. 287–303. doi:10.1007/978-3-662-43550-2_11.

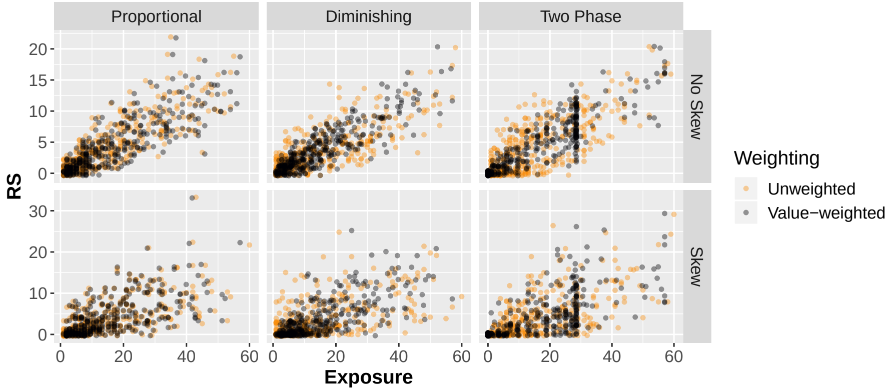

(a) Simulated RS by age data. Orange points are unweighted years lived. Black points are effective years lived after value-weighting using the age-specific RS functions in frames (b) or (c).

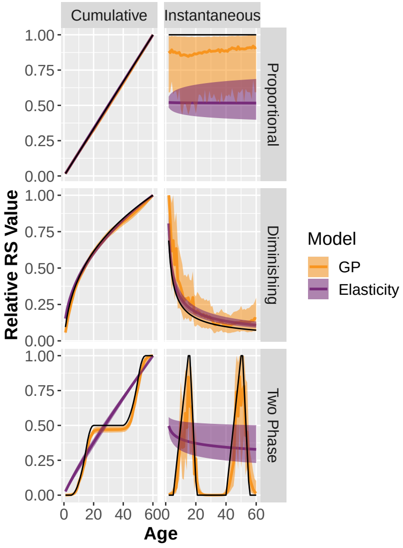

(b) Age-specific RS curves (data, no skew).

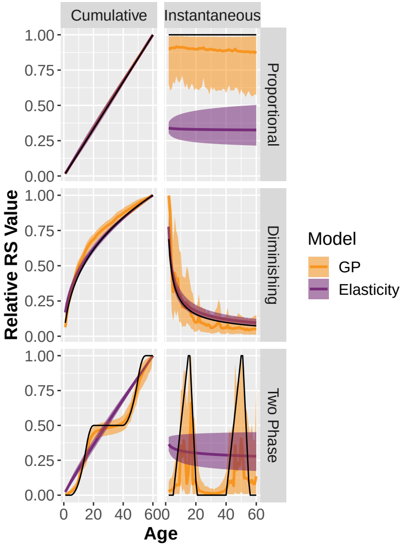

(c) Age-specific RS curves (data, skew).

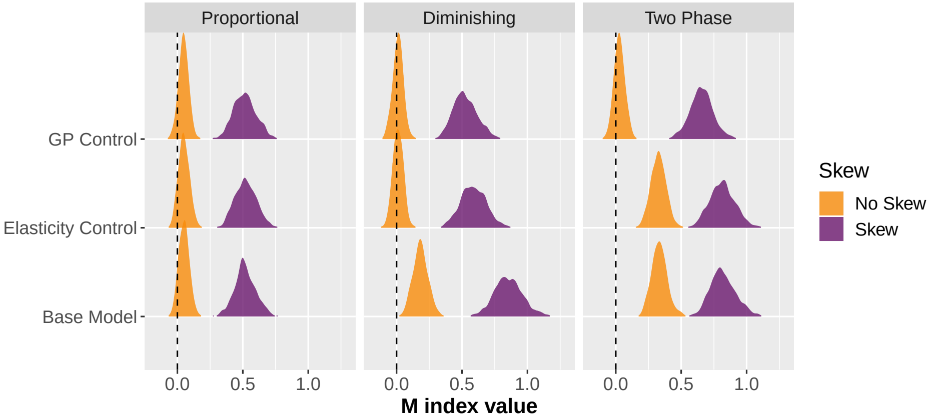

(d) Estimates of M from each data set using each of the three versions of age-adjustment.

Figure 1: In frame (a) we plot simulated RS by age data. We generate simulated RS data as a function of age using three models (proportional returns, diminishing returns, and highly non-linear returns) for each of two skew values (no skew—based on Poisson residuals, and high skew—based on over-dispersed, Negative Binomial residuals). These three models are shown in frames (b) and (c). The black curves in frames (b) and (c) are the curves used to generate the simulated data; these curves were identical in the generative model of RS without skew (frame b) and with skew (frame c). Estimates of these same curves are plotted using elasticity control (in purple) and GP control (in orange) for each data set. Because exposure time data is only modeled up to a proportion, only the shape, not the y-axis location of the function should be evaluated here. If RS is proportional to age, then the basic M metric, the M metric with elasticity control, and the M metric with GP control, all recover the correct form of the value function, and generate equivalent estimates in frame (d). If marginal RS is diminishing with age, then the basic M metric is upwardly biased, but the M metric with either elasticity control or GP control recovers the correct form of the value function and produces correct estimates in frame (d). If RS is a highly non-linear function of age—as it is in the two-phase function plotted in frames (b) and (c)—then both the standard M metric and the M metric with elasticity control fail to recover the correct form of the value function; the M metric with GP control, however, still recovers the correct form of the value function and produces correct estimates in frame (d).

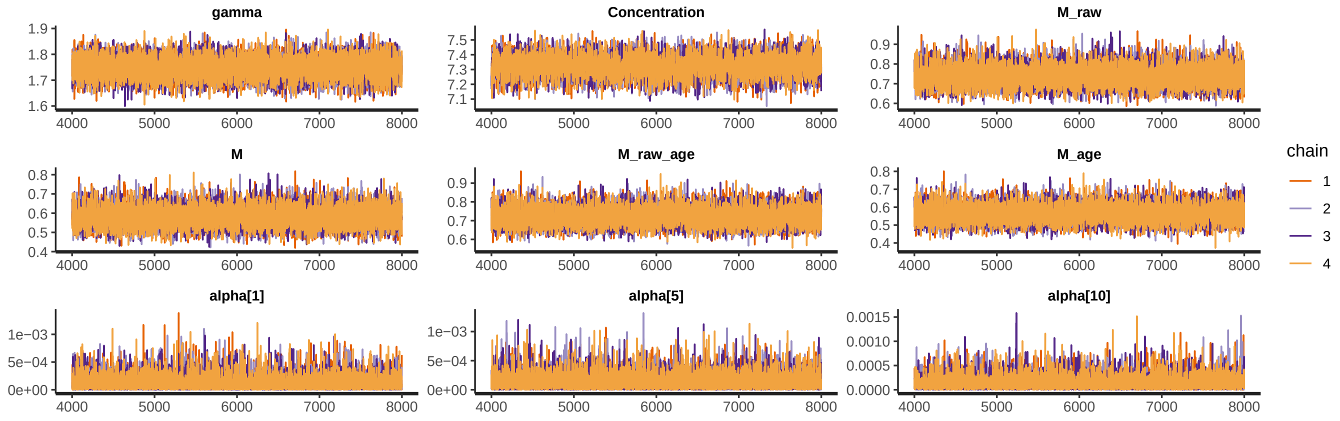

(a) Males.

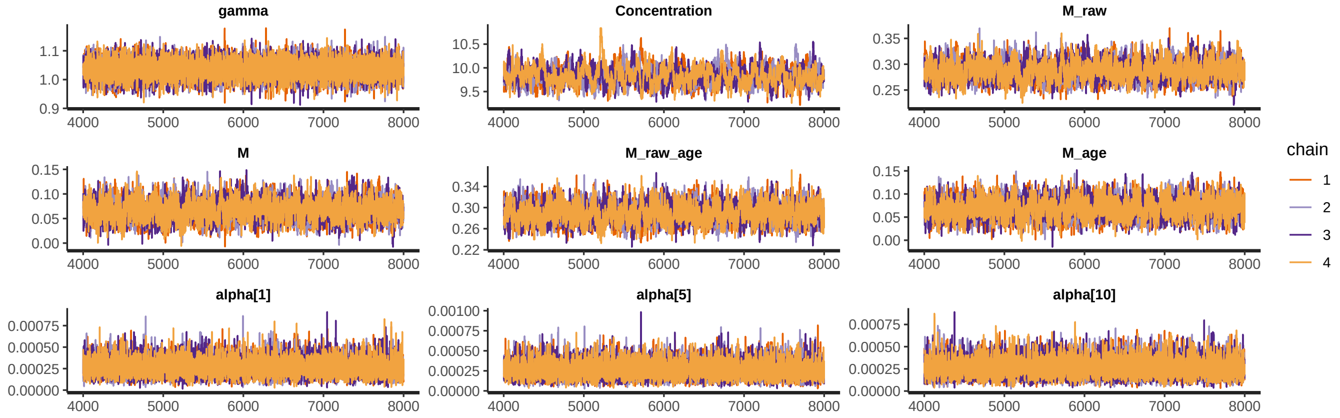

(b) Females.

Figure 2: Traceplots of parameters underlying M estimates in the Kipsigis data.

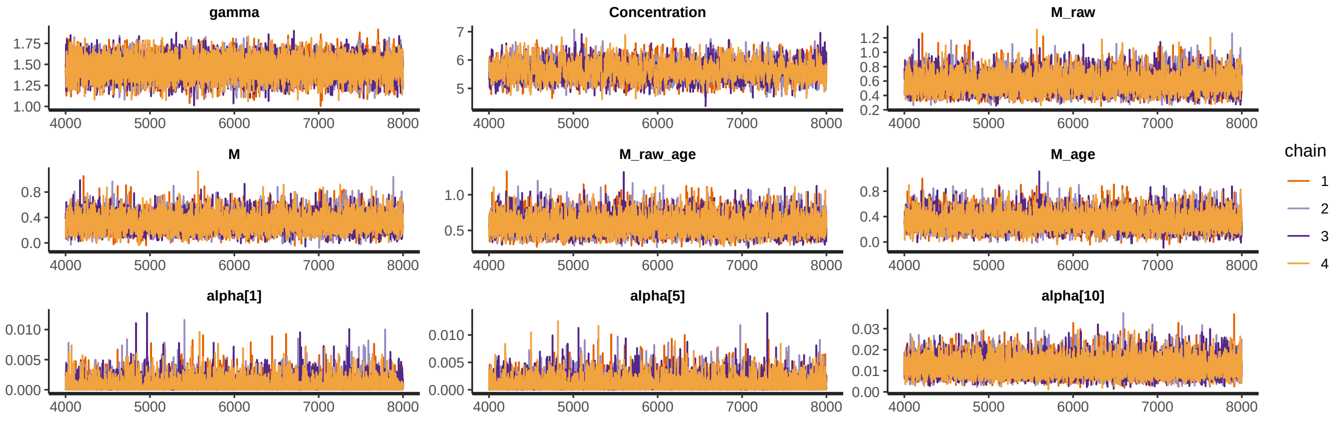

(a) Males.

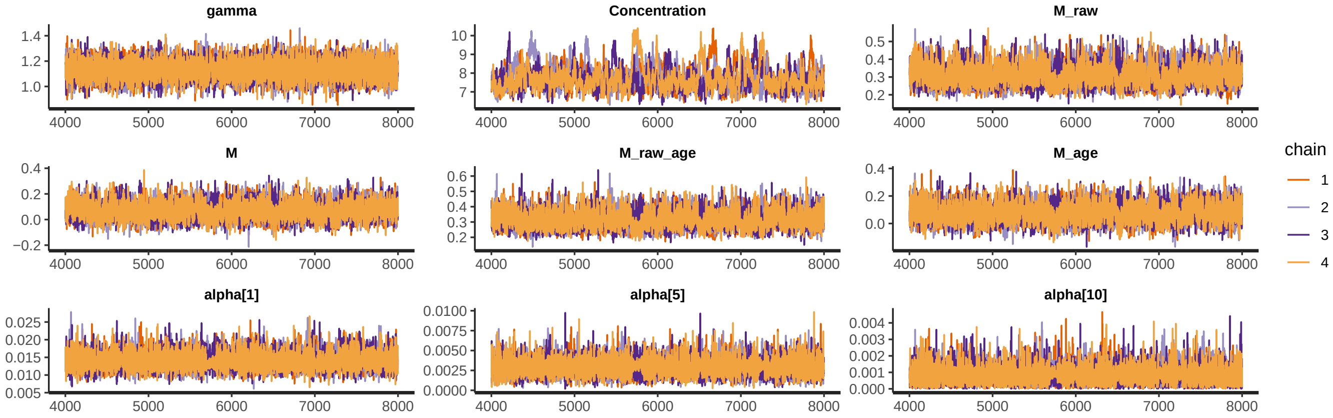

(b) Females.

Figure 3: Traceplots of parameters underlying M estimates in the Afrocolombian data.

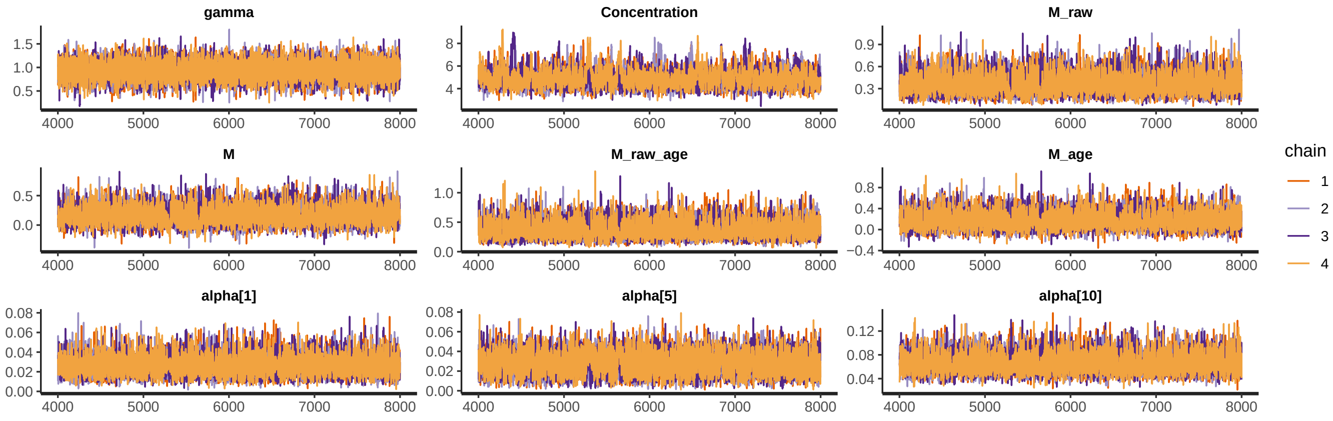

(a) Males.

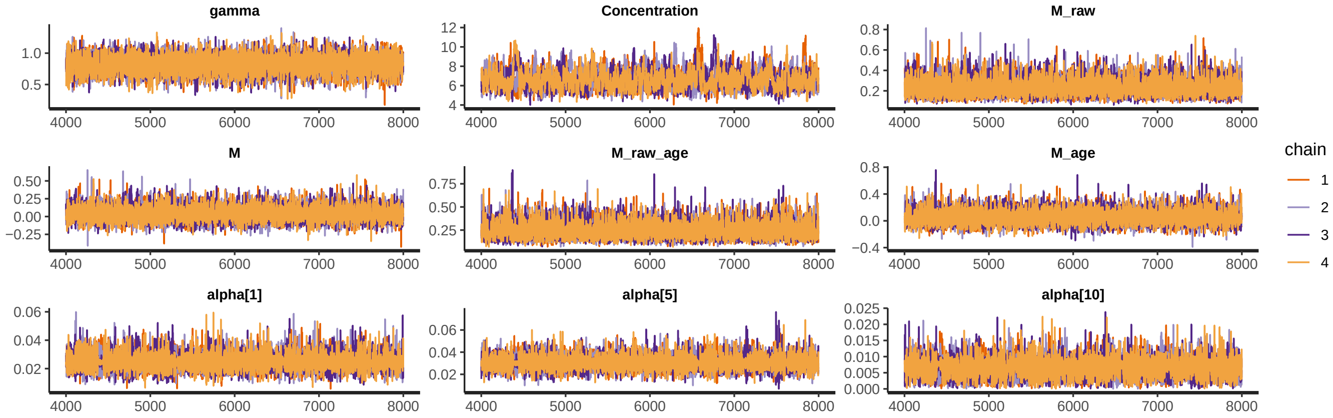

(b) Females.

Figure 4: Traceplots of parameters underlying M estimates in the Emberá data.

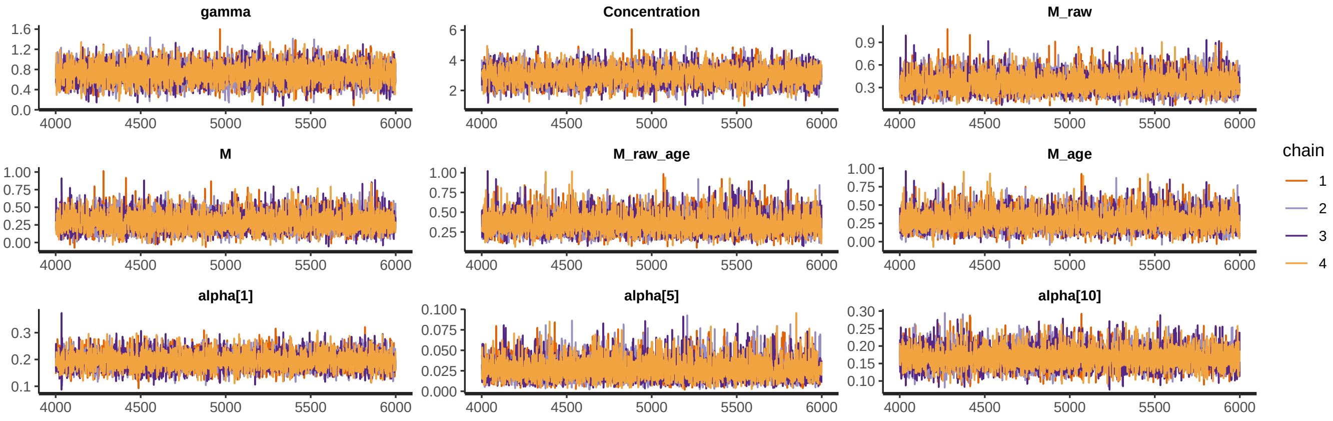

(a) N=10.

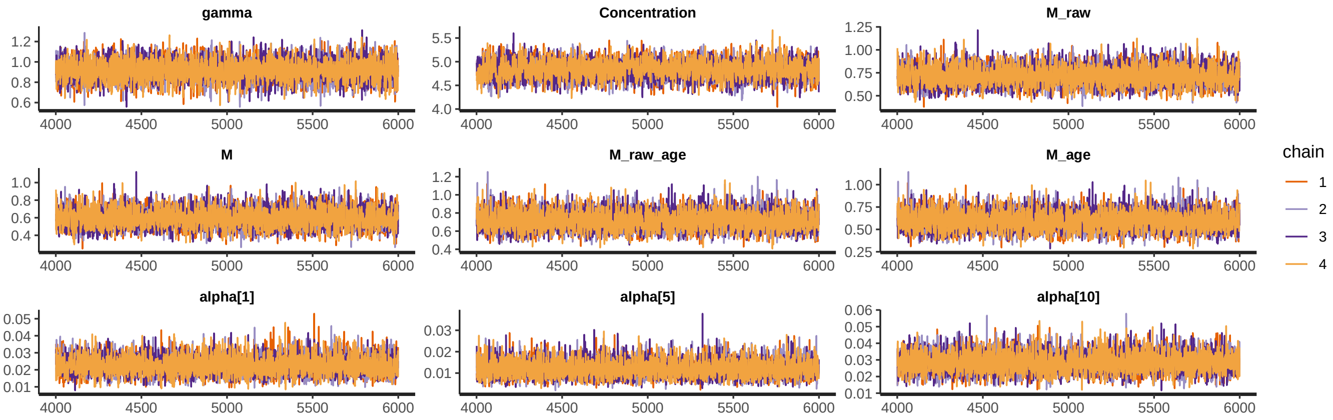

(b) N=80.

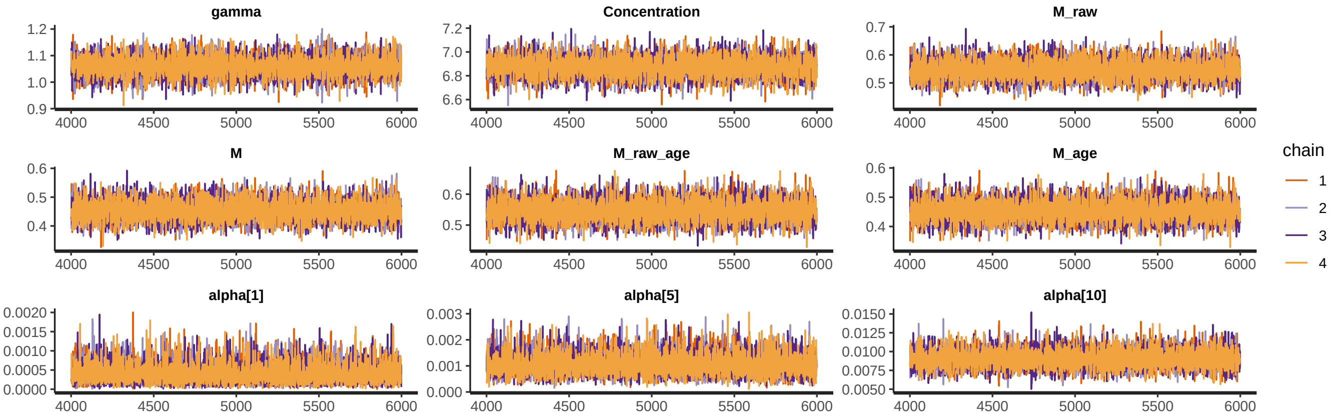

(c) N=450.

Figure 5: Traceplots of parameters underlying M estimates in the central panel in the main text Figure 3, with mid-level skew and mid-level mean RS, for three values of sample size.

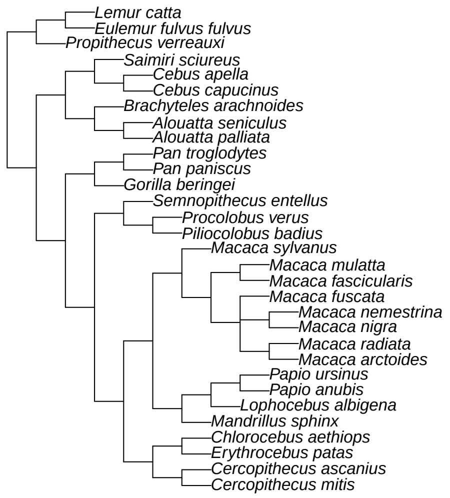

(a) 10ktrees phylogeny with unit branch lengths.

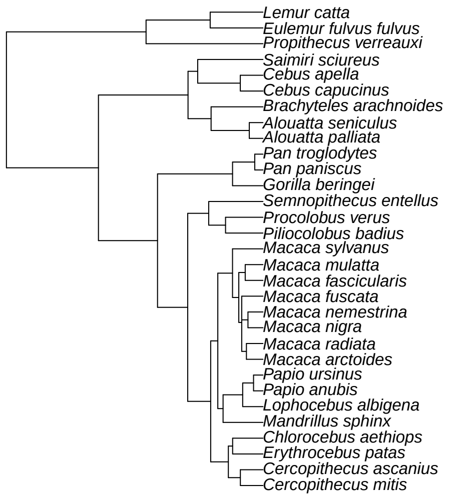

(b) 10ktrees phylogeny with numerical branch lengths.

Figure 6: Phylogenetic data used in our analyses.

(a) Model of M

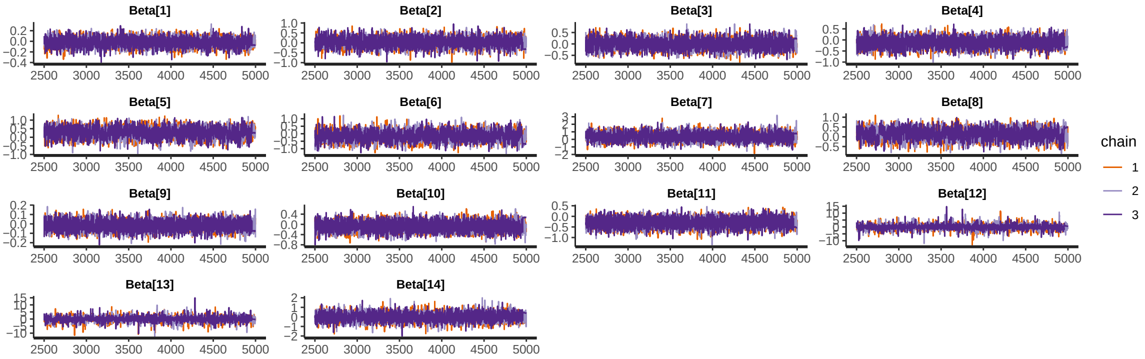

(b) Model of B

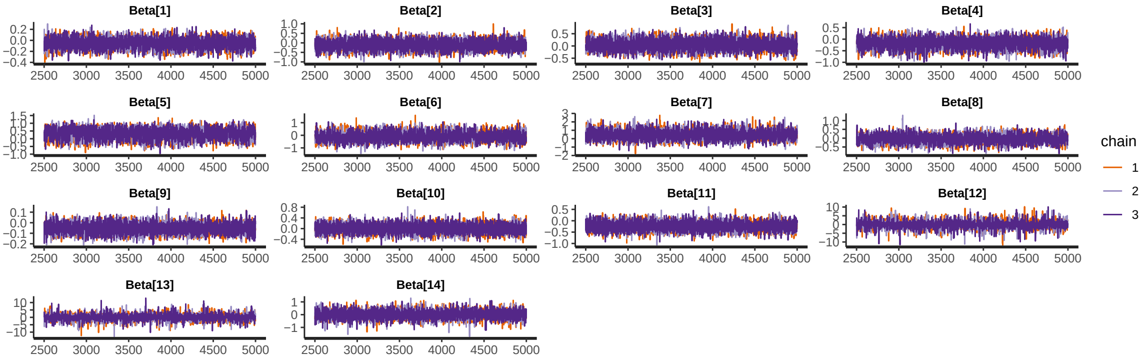

(c) Model of λ

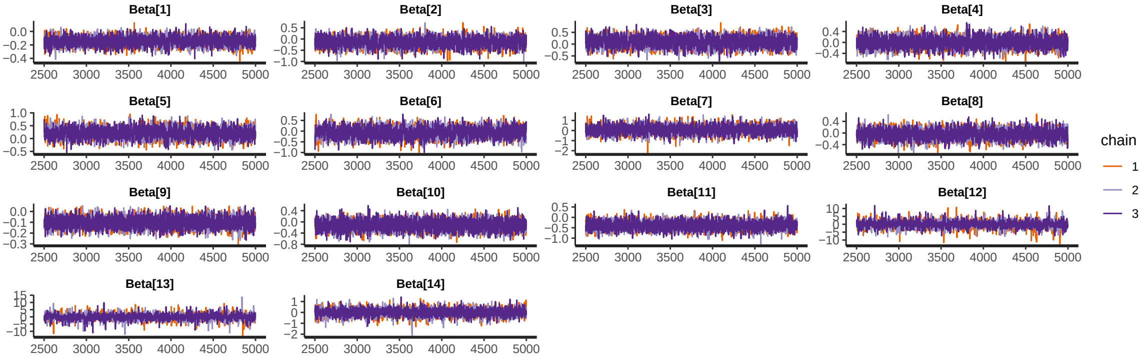

(d) Model of MMP

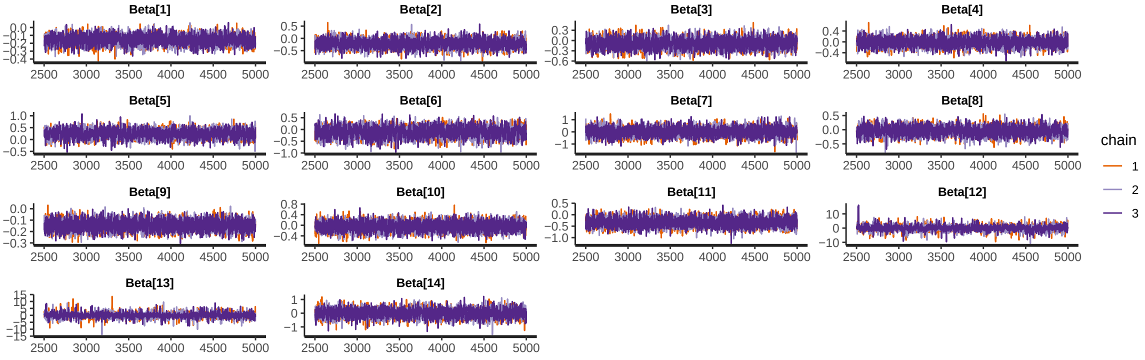

Figure 7: Traceplots of parameters from the multivariate phylogentic mixed-effects models.

Table 1: Univariate associations following K&N Table 1.

Variable	Metric	Slope	SE	DF	T	P
N males	Lambda	-0.79	0.13	25	-5.99	<**0.01**
N females	Lambda	-0.46	0.14	24	-3.33	<**0.01**
N copulations	Lambda	-0.2	0.1	22	-1.95	0.06
Breed. Seas.	Lambda	0.03	0.19	22	0.15	0.88
Dur. estrous	Lambda	0.13	0.14	20	0.88	0.39
Exp. estr. overlap	Lambda	0	0.07	20	-0.01	1
Obs. estr. overlap	Lambda	0	0.01	19	-0.09	0.93
Synchrony	Lambda	-0.08	0.38	12	-0.22	0.83
Male dispersal	Lambda	-0.36	0.25	3	-1.46	0.24
N males	MMP	-0.77	0.12	28	-6.59	<**0.01**
N females	MMP	-0.34	0.14	27	-2.45	**0.02**
N copulations	MMP	-0.25	0.06	22	-3.91	<**0.01**
Breed. Seas.	MMP	-0.01	0.17	23	-0.07	0.95
Dur. estrous	MMP	0.12	0.13	21	0.92	0.37
Exp. estr. overlap	MMP	0.01	0.07	21	0.12	0.91
Obs. estr. overlap	MMP	0	0	20	-0.43	0.67
Synchrony	MMP	-0.3	0.32	13	-0.92	0.37
Male dispersal	MMP	-0.57	0.14	2	-4.14	**0.05**
N males	B	-0.1	0.1	20	-1	0.33
N females	B	-0.13	0.05	19	-2.4	**0.03**
N copulations	B	0	0.04	18	-0.1	0.92
Breed. Seas.	B	-0.11	0.07	16	-1.57	0.14
Dur. estrous	B	0.02	0.05	14	0.43	0.67
Exp. estr. overlap	B	0	0.02	14	0.11	0.92
Obs. estr. overlap	B	0	0	13	0.95	0.36
Synchrony	B	-0.02	0.13	8	-0.12	0.91
Male dispersal	B	0.04	0.07	1	0.59	0.66
N males	M	-0.28	0.19	23	-1.44	0.16
N females	M	-0.14	0.17	22	-0.85	0.4
N copulations	M	-0.02	0.11	19	-0.19	0.85
Breed. Seas.	M	-0.41	0.25	20	-1.63	0.12
Dur. estrous	M	0.12	0.14	18	0.81	0.43
Exp. estr. overlap	M	0.09	0.1	17	0.86	0.4
Obs. estr. overlap	M	0	0.01	17	-0.1	0.92
Synchrony	M	0.18	0.44	11	0.4	0.7
Male dispersal	M	0.21	0.37	3	0.57	0.61

Table 2: Univariate associations with the sample restricted to species for which all four skew indices were available.

Variable	Metric	Slope	SE	DF	T	P
N males	Lambda	-0.78	0.12	23	-6.22	<**0.01**
N females	Lambda	-0.48	0.13	22	-3.66	<**0.01**
N copulations	Lambda	-0.16	0.11	20	-1.51	0.15
Breed. Seas.	Lambda	-0.04	0.19	20	-0.22	0.83
Dur. estrous	Lambda	0.08	0.18	18	0.44	0.67
Exp. estr. overlap	Lambda	-0.03	0.08	18	-0.31	0.76
Obs. estr. overlap	Lambda	0	0.01	15	-0.23	0.82
Synchrony	Lambda	-0.39	0.49	11	-0.8	0.44
Male dispersal	Lambda	-0.36	0.25	3	-1.46	0.24
N males	MMP	-0.74	0.09	24	-7.82	<**0.01**
N females	MMP	-0.42	0.1	22	-4.28	<**0.01**
N copulations	MMP	-0.21	0.07	19	-3.03	**0.01**
Breed. Seas.	MMP	-0.06	0.17	20	-0.37	0.71
Dur. estrous	MMP	0.07	0.15	18	0.48	0.63
Exp. estr. overlap	MMP	-0.03	0.07	18	-0.36	0.72
Obs. estr. overlap	MMP	0	0.01	15	-0.35	0.73
Synchrony	MMP	-0.37	0.4	11	-0.92	0.38
Male dispersal	MMP	-0.57	0.14	2	-4.14	**0.05**
N males	B	-0.1	0.1	20	-1	0.33
N females	B	-0.13	0.05	19	-2.4	**0.03**
N copulations	B	0	0.04	18	-0.1	0.92
Breed. Seas.	B	-0.11	0.07	16	-1.57	0.14
Dur. estrous	B	0.02	0.05	14	0.43	0.67
Exp. estr. overlap	B	0	0.02	14	0.11	0.92
Obs. estr. overlap	B	0	0	13	0.95	0.36
Synchrony	B	-0.02	0.13	8	-0.12	0.91
Male dispersal	B	0.04	0.07	1	0.59	0.66
N males	M	-0.28	0.19	23	-1.44	0.16
N females	M	-0.14	0.17	22	-0.85	0.4
N copulations	M	-0.02	0.11	19	-0.19	0.85
Breed. Seas.	M	-0.41	0.25	20	-1.63	0.12
Dur. estrous	M	0.12	0.14	18	0.81	0.43
Exp. estr. overlap	M	0.09	0.1	17	0.86	0.4
Obs. estr. overlap	M	0	0.01	17	-0.1	0.92
Synchrony	M	0.18	0.44	11	0.4	0.7
Male dispersal	M	0.21	0.37	3	0.57	0.61

Table 3: Multiple regression models following K&N Table 2.

Variable	Metric	Slope	SE	DF	T	P
N males	Lambda	-0.38	0.19	12	-2.01	0.07
N females	Lambda	-0.38	0.25	12	-1.56	0.14
N copulations	Lambda	-0.1	0.12	12	-0.83	0.42
Breed. Seas.	Lambda	0.2	0.14	12	1.46	0.17
Exp. estr. overlap	Lambda	0.06	0.06	12	0.95	0.36
Male dispersal	Lambda	0.54	0.27	12	2.01	0.07
N males	MMP	-0.46	0.16	13	-2.88	**0.01**
N females	MMP	-0.12	0.17	13	-0.69	0.5
N copulations	MMP	-0.19	0.07	13	-2.74	**0.02**
Breed. Seas.	MMP	0.07	0.1	13	0.73	0.48
Exp. estr. overlap	MMP	0.05	0.05	13	1.04	0.32
Male dispersal	MMP	0.34	0.23	13	1.52	0.15
N males	B	-0.3	0.12	9	-2.46	**0.04**
N females	B	-0.22	0.15	9	-1.45	0.18
N copulations	B	0.17	0.08	9	2.01	0.08
Breed. Seas.	B	0.03	0.08	9	0.32	0.76
Exp. estr. overlap	B	0.03	0.05	9	0.68	0.51
Male dispersal	B	0.36	0.15	9	2.31	**0.05**
N males	M	-0.79	0.46	10	-1.72	0.12
N females	M	-0.46	0.61	10	-0.75	0.47
N copulations	M	0.5	0.35	10	1.43	0.18
Breed. Seas.	M	0.01	0.35	10	0.04	0.97
Exp. estr. overlap	M	0.03	0.19	10	0.18	0.86
Male dispersal	M	1.24	0.65	10	1.9	0.09

Table 4: Multiple regressions with the sample restricted to species for which all four skew indices were available.

Variable	Metric	Slope	SE	DF	T	P
N males	Lambda	-0.48	0.17	10	-2.89	**0.02**
N females	Lambda	-0.5	0.22	10	-2.27	**0.05**
N copulations	Lambda	0.06	0.13	10	0.49	0.63
Breed. Seas.	Lambda	0.18	0.13	10	1.47	0.17
Exp. estr. overlap	Lambda	0.06	0.07	10	0.91	0.38
Male dispersal	Lambda	0.49	0.24	10	2.06	0.07
N males	MMP	-0.55	0.11	10	-4.77	**<0.01**
N females	MMP	-0.32	0.15	10	-2.1	0.06
N copulations	MMP	0.01	0.09	10	0.06	0.95
Breed. Seas.	MMP	0.08	0.09	10	0.97	0.35
Exp. estr. overlap	MMP	0.04	0.05	10	0.9	0.39
Male dispersal	MMP	0.35	0.16	10	2.13	0.06
N males	B	-0.3	0.12	9	-2.46	**0.04**
N females	B	-0.22	0.15	9	-1.45	0.18
N copulations	B	0.17	0.08	9	2.01	0.08
Breed. Seas.	B	0.03	0.08	9	0.32	0.76
Exp. estr. overlap	B	0.03	0.05	9	0.68	0.51
Male dispersal	B	0.36	0.15	9	2.31	**0.05**
N males	M	-0.79	0.46	10	-1.72	0.12
N females	M	-0.46	0.61	10	-0.75	0.47
N copulations	M	0.5	0.35	10	1.43	0.18
Breed. Seas.	M	0.01	0.35	10	0.04	0.97
Exp. estr. overlap	M	0.03	0.19	10	0.18	0.86
Male dispersal	M	1.24	0.65	10	1.9	0.09

Table 5: Intra-specific analysis: male number and female number.

Variable	Metric	Slope	SE	DF	T	P
N males	Lambda	-0.18	0.05	14	-3.44	<**0.01**
N females	Lambda	0.05	0.03	14	1.66	0.12
N males	MMP	-0.17	0.04	14	-3.74	<**0.01**
N females	MMP	0.05	0.03	14	1.7	0.11
N males	B	-0.08	0.04	9	-1.88	0.09
N females	B	0.02	0.03	9	0.9	0.39
N males	M	-0.17	0.15	9	-1.17	0.27
N females	M	0.05	0.09	9	0.51	0.62

Table 6: Intra-specific analysis: male number and expected estrous overlap.

Variable	Metric	Slope	SE	DF	T	P
N males	Lambda	-0.18	0.05	14	-3.57	<**0.01**
Exp. estr. overlap	Lambda	0.02	0.01	14	1.72	0.11
N males	MMP	-0.16	0.04	14	-3.88	<**0.01**
Exp. estr. overlap	MMP	0.02	0.01	14	1.76	0.1
N males	B	-0.08	0.04	9	-1.95	0.08
Exp. estr. overlap	B	0.01	0.01	9	0.94	0.37
N males	M	-0.17	0.14	9	-1.24	0.25
Exp. estr. overlap	M	0.02	0.04	9	0.56	0.59

Table 7: Intra-specific analysis: male number and female number, in cases where all skew indices were available.

Variable	Metric	Slope	SE	DF	T	P
N males	Lambda	-0.2	0.06	9	-3.5	**0.01**
N females	Lambda	0.07	0.03	9	1.93	0.09
N males	MMP	-0.18	0.05	9	-3.87	<**0.01**
N females	MMP	0.06	0.03	9	2.08	0.07
N males	B	-0.08	0.04	9	-1.88	0.09
N females	B	0.02	0.03	9	0.9	0.39
N males	M	-0.17	0.15	9	-1.17	0.27
N females	M	0.05	0.09	9	0.51	0.62

Table 8: Intra-specific analysis: male number and expected estrous overlap, in cases where all skew indices were available.

Variable	Metric	Slope	SE	DF	T	P
N males	Lambda	-0.19	0.05	9	-3.59	**0.01**
Exp. estr. overlap	Lambda	0.03	0.01	9	1.96	0.08
N males	MMP	-0.18	0.04	9	-3.96	<**0.01**
Exp. estr. overlap	MMP	0.02	0.01	9	2.1	0.07
N males	B	-0.08	0.04	9	-1.95	0.08
Exp. estr. overlap	B	0.01	0.01	9	0.94	0.37
N males	M	-0.17	0.14	9	-1.24	0.25
Exp. estr. overlap	M	0.02	0.04	9	0.56	0.59
